# Diffuse Myocardial Fibrosis and Cardiomyocyte Diameter Are Associated With Heart Failure Symptoms in Chagas Cardiomyopathy

**DOI:** 10.3389/fcvm.2022.880151

**Published:** 2022-06-17

**Authors:** Cristiane Nardi Gemme, Thiago Quinaglia A. C. Silva, Luiz C. Martins, Luis Miguel da Silva, Layde Rosane Paim, Andrei Sposito, Wilson Nadruz, Fabio Fernandes, Sergio San Juan Dertkigil, Jamiro da Silva Wanderley, Eros A. de Almeida, Konradin Metze, Tomas G. Neilan, Michael Jerosch-Herold, Otávio R. Coelho-Filho

**Affiliations:** ^1^Faculdade de Ciências Médicas, Universidade Estadual de Campinas, São Paulo, Brazil; ^2^Division of Cardiology, Department of Radiology, Cardiovascular Imaging Research Center, Massachusetts General Hospital, Harvard Medical School, Boston, MA, United States; ^3^Cardiomyopathy Unit, Heart Institute, University of São Paulo, São Paulo, Brazil; ^4^Non-invasive Cardiovascular Imaging Program, Department of Radiology, Brigham and Women's Hospital and Harvard Medical School, Boston, MA, United States

**Keywords:** Chagas disease, cardiac magnetic resonance, interstitial fibrosis, cardiomyocyte diameter, heart failure

## Abstract

**Background:**

Chronic Chagas cardiomyopathy (CCC) constitutes the most life-threatening consequence of the *Trypanosoma cruzi* infection. Our goal was to test in CCC the associations of the myocardial tissue phenotype with cardiac dysfunction, and heart failure (HF) severity, using cardiac magnetic resonance (CMR).

**Methods:**

We performed a prospective observational cohort of patients with consecutive CCC with a CMR protocol, including ventricular function, myocardial T1, and late gadolinium enhancement (LGE). Extracellular volume (ECV), and intracellular water lifetime, τ_ic_, a measure of cardiomyocyte diameter, were compared to CCC disease progression, including Rassi score and New York Heart Association (NYHA) class. An exploratory prognostic analysis was performed to investigate the association of both ECV and τ_ic_ with CV death.

**Results:**

A total of 37 patients with intermediate-to-high-risk CCC were enrolled (Chagas Rassi score ≥7, mean left ventricle (LV) ejection fraction (EF) 32 ± 16%). Myocardial ECV (0.40 ± 0.07) was correlated with Rassi score (r = 0.43; *P* = 0.009), higher NYHA class, and LV EF (r = −0.51; *P* = 0.0015). τ_ic_ decreased linearly with NYHA class (*P* = 0.007 for non-parametric test of linear trend) and showed a positive association with LV EF (r = 0.47; *P* = 0.004). Over a median follow-up of 734 days (range: 6–2,943 days), CV death or cardiac transplantation occurred in 10 patients. The Rassi score (heart rate [HR] = 1.3; 95% CI = [1.0, 1.8]; *P* = 0.028) and ECV (HR = 3.4 for 0.1 change, 95% CI = [1.1, 11.0], *P* = 0.039) were simultaneously associated with CV death.

**Conclusion:**

In patients with intermediate-to-high-risk CCC, an expanded ECV and regression of cardiomyocyte diameter were associated with worsening systolic function and HF severity, respectively. The exploratory analysis indicates that ECV may have a prognostic value to identify patients with CCC at a higher risk for cardiovascular events.

## Introduction

The Centers for Disease Control and Prevention (CDC) declared that Chagas disease is one of the major neglected parasitic infections in the United States and other developed countries. Chagas disease has been reported to affect up to 6 million patients in South America (1) and migration patterns have resulted in a growing caseloads in developed countries, with over 300,000 patients with Chagas disease in the US (2). Moreover, according to Healthcare Cost and Utilization Project National Inpatient Sample, the number of patients with chronic Chagas cardiomyopathy (CCC) requiring hospitalization have consistently increased from 2002 through 2017 (3).

The CCC is the most serious consequence of long-term infection with the *Trypanosoma cruzi* protozoan and it may lead to heart failure (HF), stroke, arrhythmias, and even sudden cardiac death. Between 20 and 40% of those infected with the protozoan will develop CCC (1) and, compared to other cardiomyopathies, the prognosis with CCC is generally worse (4–6). Thus, there is a need for methods to improve myocardial characterization and risk stratification among patients with CCC. A recent joint expert consensus by the Brazilian Cardiovascular Imaging Association and the European Association of Cardiovascular Imaging suggested a key role for the detection and quantification of replacement fibrosis by late gadolinium enhancement (LGE) using cardiac magnetic resonance (CMR) as an essential tool for risk stratification among patients with CCC (7). This recommendation is based on a series of previous studies showing the prognostic impact of LGE (8–10). Focal fibrosis, assessed by LGE, in individuals with established CCC is detected in about 70–90% and this increases to 100% in the presence of left ventricle (LV) systolic dysfunction (8, 11). CMR techniques for tissue phenotyping have expanded beyond LGE and include the measurement of the extracellular volume (ECV) fraction for assessing diffuse fibrosis, and estimation of the intracellular water-lifetime, to assess cardiomyocyte diameter (12). However, there are limited data on the role of diffuse myocardial fibrosis and cardiomyocyte remodeling among patients with CCC. Diffuse interstitial fibrosis has consistently been observed in histological studies alongside replacement fibrosis in CCC (13, 14). Therefore, in this study, we aimed to investigate, in a cohort of patients with intermediate-to-high-risk CCC, the clinical significance of diffuse myocardial fibrosis and cardiomyocyte remodeling and their associations with global ventricular remodeling, disease severity, and clinical outcomes.

## Methods

### Subjects

We performed a prospective observational study for which consecutive patients with two successive, serologically positive tests for Chagas and diagnosis of CCC were recruited from the Cardiomyopathies Outpatient Clinic at the University of Campinas (Campinas—São Paulo, Brazil). The criteria for CCC diagnosis were the presence of any of the following: (1) atrioventricular block (PR interval > 0.2 s), left or right bundle branch block, left anterior fascicular block, premature ventricular beats, or abnormal Q waves; (2) cardiomegaly on chest radiography; (3) abnormal LV systolic function (LV ejection fraction <50%), or any wall motion abnormality; and (4) ventricular tachycardia on ECG monitoring. The Rassi score for death in CCC was obtained in all patients (15). Briefly, this score is composed of the following risk markers: New York Heart Association (NYHA) class III or IV, evidence of cardiomegaly on radiography, LV systolic dysfunction on echocardiography, non-sustained ventricular tachycardia on 24-h Holter monitoring, low QRS voltage on electrocardiography, and male sex. Three categories of risk have originally been described for the Rassi score: low (0–6 points on the Rassi score), intermediate (7–11 points), and high (12–20 points). All patients gave written informed consent after reading the information about the study by signing the consent form. The study protocol was approved by the local institutional review board (CAAE: 2647481920005404).

### Cardiac Magnetic Resonance

All patients underwent CMR imaging in a 3 Tesla system (Achieva, Philips Medical Systems, Best, The Netherlands) with a 6-element phased-array surface coil. The CMR sequences were ECG-gated and followed our routine protocol for LV function, viability assessment, and tissue characterization in patients with CCC. Cine imaging with steady-state free precession was performed in short-axis and long-axis-views to assess LV mass and volumes (8-mm thickness without interslice gap in short-axis stack, repetition time: 3.4 ms, echo time: 1.2 ms, and in-plane spatial resolution 1.5 mm). LGE images were obtained in slices matching those for cine imaging with a segmented, phase-sensitive inversion-recovery-prepared (PSIR) gradient-echo acquisition, triggered every other heartbeat at approximately 10 min after a cumulative gadolinium dose of 0.2 mmol/kg (gadoterate meglumine; Dotarem, Guerbet, Aulnay-sous-Bois, France). The presence of LGE in the LV in the PSIR images was quantified as a percentage of total LV mass using 5SD criteria. T1 measurements for ECV and intracellular water lifetime (τ_*ic*_) were performed with an inversion-recovery cine Look-Locker sequence with segmented gradient-echo acquisition (echo time 2.7 ms, TR 5.5 ms, flip angle 10°, matrix size 192 × 128, slice thickness 8 mm), covering approximately 2 cardiac cycles (inversion time increments: 100 ms pre-contrast and 55 ms post-contrast, slice thickness 8 mm, TR>4 RR intervals pre-contrast and 3 RR intervals post-contrast). The T1 imaging was repeated in the same mid-LV short-axis slice, before and 5–7 times after the injection of gadolinium. Clinical, anthropometric, and hemodynamic data were obtained at the same CMR exam. Only euvolemic ambulatory patients, judged to be likely to tolerate a 50-min CMR in a supine position and not receiving IV therapy (e.g., diuretics or inotropes), were recruited for imaging.

### Quantification of Extracellular Volume Fraction and Cardiomyocyte Size (τ_ic_)

The MASS CMR software (Mass Research, Leiden University Medical Center, Leiden, The Netherlands) was used for the analysis of images from T1 images. The LV wall was divided into 6 standard segments, and inversion recovery curves were generated for each segment. The segmental T1^*^ was determined by a non-linear least-squares fitting to an analytic expression for the inversion recovery. T1 was obtained from T1^*^ by correction for the effects of the Look-Locker readout during the inversion recovery. Then, myocardial R1 (${\text{R}}_{1}^{\text{myocardial}}$) was fit as a function of blood R1 (${\text{R}}_{1}^{\text{blood}}$) with a 2-compartment model of transcytolemmal water exchange to determine ECV and the intracellular water lifetime, as previously described (12). The model equation for 2-site water exchange is shown in Supplementary Figure 1, together with an example of a fit to determine the intracellular water lifetime. Segments with LGE were excluded from the quantification of mean LV ECV.

### Statistical Analysis

Statistical analysis was performed using R version 4.0.3 (released 10 October 2020; R Foundation for Statistical Computing, Vienna, Austria). Data are presented as mean ± standard deviation if normally distributed, as median with interquartile range (IQR) if not normally distributed, or as count (percentage) for categorical variables. A *t*-test was used for the comparison of normally distributed values and, otherwise, the Mann–Whitney test for continuous variables. Associations between variables were assessed by the linear regression analysis and the Pearson correlation coefficient. Differences in correlation strength for two correlations sharing one common variable were tested using Steiger's method. Specifically, we were interested in comparing the correlation strengths of tissue markers with systolic function and ventricular remodeling, respectively. To test for a linear trend of a continuous CMR variable with ordered levels of NYHA class, we used the non-parametric Jonckheere–Terpstra (J-T) test. The primary outcome of interest for the exploratory survival analysis was CV death, and heart transplantation was considered a competing risk. We defined follow-up time as days from the date of CMR (entry time) to the date for the last follow-up or the date of death or cardiac transplantation. Cumulative incidence functions (CIFs) were calculated for each CV death and heart transplantation, and stratified by the median ECV, intracellular water lifetime, native T1 and Rassi score, respectively, to determine the effect of these on outcomes. The subdistributions for a competing risk were compared across strata using Gray's test implemented in the R-package “cmprsk”(16). Univariable and multivariable associations of risk covariates with CV death were determined by Cox proportional hazards regression with heart transplant as a competing risk.

## Results

Tables 1–3 summarize the demographics, clinical characteristics, CMR parameters, and ECG data, respectively, of the study population, and two subgroups stratified by the median ECV (0.399) at baseline. A total of 37 patients (mean age of 53 [47, 61] years, mean body surface area of 1.71 ± 0.20 m^2^, and 40.5% were female) with intermediate-to-high-risk CCC as determined by the Rassi score (15) (≥7, mean 10 [8, 13.5]) (Tables 1, 4) were enrolled between July 2012 and July 2020, with the majority showing clinical symptoms for HF (76% had NYHA >/=II). Right bundle block and non-sustained ventricular tachycardia were observed in 18 and 14 patients, respectively (49 and 37%) (Table 3). Patients with CCC were under adequate HF therapy in agreement with recent recommendations (17, 18), with all patients receiving either angiotensin-converting enzyme inhibitor (62%, *n* = 23) or angiotensin receptor blocker (38%, *n* = 14), and >80% being treated with beta blocker (83%, *n* = 31), as summarized in Table 1 (“medications”). While the prior history of angina was present in 5% (*n* = 2), significant coronary artery disease by coronary angiography (CA), defined as ≥70% narrowing of luminal diameter of any of the three major epicardial arteries, and assessed by invasive CA (ICA) in the majority of patients with CCC (89%, *n* = 33), was observed in only one patient. In the remaining 4 patients without ICA, CAD was ruled out by functional tests. Among the 35 (91%) patients of our CCC cohort with positive scar detected for LGE, none had typical findings for ischemic heart disease, excluding the possibility of overlapping ischemic heart disease (Table 2). Moreover, specific types of cardiomyopathies were also excluded by clinical history, and all available diagnostic test results, including the CMR findings.

**Table 1:** General characteristics of enrolled patients.

	**All patients**	**ECV < median**	**ECV ≥median**	***P*** **-value**
	**(*n* = 37)**	**(*n* = 18)**	**(*n* = 19)**	
**Demographics**				
Age at CMR [years]	53.00 (47.00, 61.00)	51.50 (47.25, 62.50)	56.00 (45.50, 60.00)	*P* = 0.869
Female, *n* (%)	15.00 (40.54%)	8 (44.44%)	7 (36.84%)	*P* = 0.900
History Chagas [in years]^*^	9 (5.00, 15.00)	10.50 (7.25, 17.75)	5.00 (4.50, 12.00)	*P* = 0.131
Height [cm]	162.97 ± 7.80	161.33 ± 7.81	164.53 ± 7.68	*P* = 0.218
Weight [kg]	686.87 ± 14.97	68.34 ± 16.58	65.47 ± 13.57	*P* = 0.570
BSA [m^2^]	1.71 ± 0.20	1.71 ± 0.21	1.71 ± 0.20	*P* = 0.977
**Comorbidities**				
History of diabetes, *n* (%)	7 (18.92%)	3 (16.67%)	4 (21.05%)	*P* = 1.000
History of hypertension, *n* (%)	17 (45.95%)	8 (44.44%)	9 (47.37%)	*P* = 1.000
History of high cholesterol, *n* (%)	11 (29.73%)	4 (22.22%)	7 (36.84%)	*P* = 0.476
History of stroke, *n* (%)	1 (2.70%)	0 (0.00%)	1 (5.26%)	*P* = 1.000
History of angina, *n* (%)	2 (5.41%)	2 (11.11%)	0 (0.00%)	*P* = 0.230
History of AF, *n* (%)	3 (8.11%)	3 (18%)	0, (0%)	*P* = 0.800
**Laboratory analysis**				
Hemoglobin, g/dL	13.54 ± 1.62	13.94 ± 1.74	13.19 ± 1.45	*P* = 0.176
Hematocrit [%]	40.70 ± 4.53	41.93 ± 4.59	39.59 ± 4.30	*P* = 0.126
Creatinine, mg/dL	1.10 ± 0.31	1.10 ± 0.38	1.11 ± 0.24	*P* = 0.717
Sodium, mmol/L	140.38 ± 9.65	143.20 ± 13.81	138.16 ± 3.24	*P* = 0.169
Potassium, mmol/L	4.68 ± 0.59	4.61 ± 0.69	4.74 ± 0.50	*P* = 0.464
Glomerular filtration rate, mL/min/1.73 m^2^	74.97 ± 2331	77.00 ± 27.27	73.16 ± 19.70	*P* = 0.635
Total cholesterol, mg/dL	167.48 ± 40.31	170.40 ± 39.18	165.06 ± 42.20	*P* = 0.709
Triglycerides, mg/dL	107.12 ± 40.95	106.27 ± 29.89	107.83 ± 49.19	*P* = 0.911
LDL-cholesterol, mg/dL	99.68 ± 39.96	103.57 ± 43.44	96.47 ± 37.90	*P* = 0.636
HDL-cholesterol, mg/dL	54.61 ± 25.24	57.60 ± 29.99	50.28 ± 20.80	*P* = 0.432
Glucose, mg/dL	107.85 ± 40.16	107.73 ± 29.78	107.95 ± 47.61	*P* = 0.987
**Clinical characteristics**				
NYHA class I, *n* (%)	9 (24.32%)	6 (33.33%)	3 (15.79%)	*P* = 0.044^**^
NYHA class II, *n* (%)	10 (27.03%)	7 (38.89%)	3 (15.79%)	
NYHA class III, *n* (%)	9 (24.32%)	1 (5.56%)	8 (42.11%)	
NYHA class IV, *n* (%)	9 (24.32%)	4 (22.22%)	5 (26.32%)	
Rassi score	10.00 (8.00, 13.50)	8.00 (6.00, 13.00)	12.00 (9.50, 15.00)	*P* = 0.074
**Hemodynamic data**				
Systolic blood pressure (resting), mmHg	111.75 ± 25.26	124.53 ± 23.99	100.32 ± 20.89	*P* = 0.003
Diastolic blood pressure (resting), mmHg	71.86 ± 14.45	76.06 ± 16.28	68.11 ± 11.78	*P* = 0.107
Heart rate [bpm]	70.30 ± 15.48	68.06 ± 17.27	72.42 ± 13.70	*P* = 0.402
**Medications**				
Aspirin, *n* (%)	14 (37.84%)	6 (33.33%)	8 (42.11%)	*P* = 0.737
Calcium channel blockers, *n* (%)	1 (2.7%)	0 (0.00%)	1 (5.26%)	*P* = 1.000
*B*-Blocker, *n* (%)	31 (83.78%)	16 (88.89%)	15 (78.95%)	*P* = 0.660
Diuretics, *n* (%)	18 (49.65%)	11 (61.11%)	7 (36.84%)	*P* = 0.487
Angiotensin receptor blocker, *n* (%)	14 (38.84%)	9 (50.00%)	5 (26.32%)	*P* = 0.184
Angiotensin-converting enzyme inhibitor, *n* (%)	23 (62.16%)	10 (55.56%)	13 (68.42%)	*P* = 0.508
Statin, *n* (%)	11 (29.73%)	5 (27.78%)	6 (31.58%)	*P* = 0.890
Insulin, *n* (%)	2 (5.41%)	2 (11.11%)	2 (10.53%)	*P* = 1.000
Oral diabetic medication, *n* (%)	4 (10.81%)	2 (11.11%)	2 (10.53%)	*P* = 1.000
Amiodarone, *n* (%)	15 (40.54%)	9 (50.00%)	6 (31.58%)	*P* = 0.325
Warfarin, *n* (%)	11 (29.73%)	8 (44.44%)	3 (15.79%)	*P* = 0.146
Spironolactone, *n* (%)	12 (32.43%)	7 (38.89%)	5 (26.32%)	*P* = 0.495

*Variables are summarized as mean ± SD if normally distributed, median (IQR) if not normally distributed, of as number (percentage) for categorical variables. CMR, cardiac magnetic resonance; BSA, body surface area; MI, myocardial infarction; NYHA, New York Heart Association functional classification; EDV, end diastolic volume; ESV, end systolic volume; LV, left ventricle; LGE, late gadolinium enhancement*.

* *Time since the initial diagnosis based on clinical history*.

** *Linear trend of NYHA class across ECV categories*.

**Table 2:** Cardiac magnetic resonance imaging data.

	**All patients**	**ECV < median**	**ECV ≥median**	***P*** **-value**
	**(*n* = 37)**	**(*n* = 18)**	**(*n* = 19)**	
Apical aneurysms, *n* (%)	12 (32%)	5 (27.78%)	7 (36.84%)	*P* = 0.728
Myocardial edema on T2, *n* (%)	7 (19%)	4 (22.22%)	3 (15.79%)	*P* = 0.693
LV wall motion abnormality, *n* (%)	26 (70%)	13 (72.22%)	13 (68.42%)	*P* = 1.000
LA dimension, mm	48.79 ± 12.68	47.73 ± 9.44	49.79 ± 15.34	*P* = 0.624
LA volume index, ml/m^2^	56.11 ± 35.12	45.82 ± 18.76	65.86 ± 43.91	*P* = 0.081
LVEDV index, ml/m^2^	147.01 ± 68.89	120.67 ± 46.64	171.97 ± 78.01	*P* = 0.021
LVESV index, ml/m^2^	106.88 ± 65.47	77.51 ± 44.02	134.70 ± 71.15	*P* = 0.006
LV mass index, g/m^2^	80.53 ± 28.94	74.41 ± 28.83	86.34 ± 28.59	*P* = 0.215
Mass/EDV [g/ml]	0.61 ± 0.22	0.67 ± 0.27	0.54 ± 0.14	*P* = 0.086
LV ejection fraction [%]	31.74 ± 16.14	39.06 ± 16.59	24.80 ± 12.51	*P* = 0.006
Presence of LGE, *n* (%)	92% (34)	94% (17)	89% (17)	*P* = 0.765
LV LGE % (5SD), [IQR]	12 [6, 19]	15 [0.09, 0.20]	09 [0.05, 0.18]	*P* = 0.273
Native T1 [ms]	1256.93 ± 104.66	1239.97 ± 118.96	1276.89 ± 95.49	*P* = 0.307
ECV	0.40 ± 0.07	0.35 ± 0.04	0.47 ± 0.06	*P* < 0.001
LV cardiomyocyte mass index [g/m^2^]	46.83 ± 16.14	48.14 ± 18.06	45.58 ± 14.48	*P* = 0.639
Intracellular water lifetime [s]	0.16 ± 0.05	0.17 ± 0.06	0.15 ± 0.04	*P* = 0.192

*Variables are summarized as mean ± SD if normally distributed, as median [IQR] if not normally distributed, or as number (percentage) for categorical variables. LA, left atrium; LV, left ventricle; EDV, end diastolic volume; ESV, end systolic volume; LGE, late gadolinium enhancement*.

**Table 3:** 12-lead electrocardiogram and 24-holter data.

	**All patients**	**ECV < median**	**ECV ≥median**	***P*** **-value**
	**(*n* = 37)**	**(*n* = 18)**	**(*n* = 19)**	
QRS axis, degrees	−22.42 ± 73.30	−30.4164.08	−15.26 ± 81.75	*P* = 0.538
QRS duration, ms	0.13 ± 0.04	0.14 ± 0.03	0.13 ± 0.05	*P* = 0.305
QTc, ms	0.41 ± 0.05	0.41 ± 0.05	0.41 ± 0.05	*P* = 0.936
Left bundle branch block (LBBB), *n* (%)	5 (14%)	1 (5.88%)	4 (21.05%)	*P* = 0.342
Right bundle branch block (RBBB), *n* (%)	18 (49%)	9 (52.94%)	9 (47.37%)	*P* = 1.000
Low voltage on 12-lead ECG, *n* (%)	1 (2.6%)	0 (0%)	1 (1%)	*P* = 0.543
Non-sustained ventricular tachycardia (24-holter), *n* (%)	14 (37%)	7 (38%)	7 (37%)	*P* = 0.827

**Table 4:** Components of the Rassi risk score.

**Components of Rassi risk score (pts toward score)**	***N***	**Percentage (%)**
NYHA III or IV (5 pts)	19	48.7
Cardiomegaly (5 pts)	27	69.2
Non-sustained ventricular tachycardia on 24-holter (3 pts)	14	37.9
Segmental or global WMA (3 pts)	25	64.1
Low QRS Voltage on 12-lead ECG (2 pts)	1	2.6
Male gender (2 pts)	24	61.5

The mean LV EF was 32 ± 16%, and both LV and left-atrial volume indices were markedly elevated [LV end diastolic volume (EDV) index: 147 ± 69 ml/m^2^, LV end systolic volume (ESV) index: 107 ± 66 ml/m^2^, LA volume index: 56 ± 35 ml/m^2^] compared to published normal ranges (19). While LV EF was significantly decreased in patients with CCC with ECV ≥ median (LV EF with ECV < median of 0.4: 39.06 ± 16.59% vs. ECV ≥ median: 24.80 ± 12.5%, *P* = 0.0061, Table 2), both EDV and ESV indexes were larger (LV EDV index with ECV < median of 0.4: 120.67 ± 46.64 ml/m^2^ vs. ECV ≥ median: 171.97 ± 78.01 ml/m^2^, *P* = 0.0208, and LV ESV index with ECV < median: 77.51 ± 44.02 ml/m^2^ vs. ECV ≥ median: 134.70 ± 71.15, *P* = 0.0060, Table 2). Apical aneurysms were noted in 12 patients (32%), and myocardial scar, by LGE, with an atypical pattern for coronary artery disease was detected in 34 patients (92%).

### CMR Tissue Phenotyping

**Figure 1:**
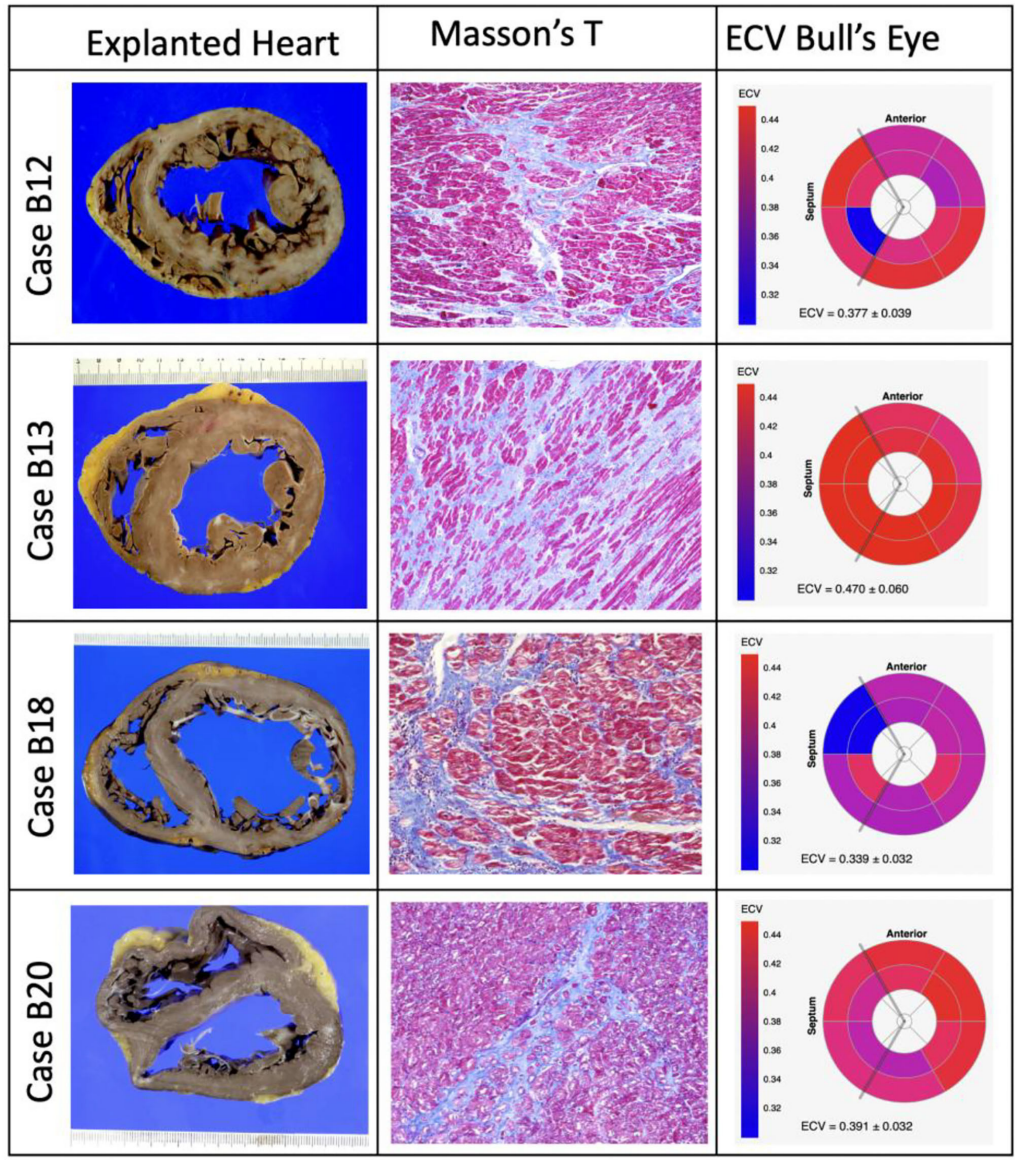
Explanted hearts (left columns), illustrative specimens of histology stained with Masson's Trichrome (middle columns), and bullseye plots of ECV from cardiac magnetic resonance (right columns) of patients who required cardiac heart transplantation with available histologic analysis.

A total of 34 (92%) patients had LGE in the LV. The percentage of LGE in the LV had a median of 12% (interquartile range: 6–19%) applying the 5SD criterion. The most commonly seen LGE pattern was epicardial, occurring exclusively in 53% (*n* = 18) of cases, epicardial in association with the mesocardial pattern in 24% (*n* = 8), and epicardial in association with mesocardial and transmural patterns in 3% (*n* = 1) of the cases (Supplementary Table 1). Lateral segments, both exclusively and in association with other regions, were the most common segments with the myocardial scar by LGE (Supplementary Table 2). Myocardial edema assessed by T2-weighted images was observed in 7 (19%) of the patients with CCC. Native T1 averaged 1,257 ± 105 ms. The LV ECV averaged 0.40 ± 0.07, which is substantially higher than the values reported for normal volunteers (20). The intracellular water lifetime averaged 0.16 ± 0.05 s. The total LV cardiomyocyte mass index, estimated from LV mass index and ECV as LV mass index, multiplied by (1-ECV), averaged 46.8 ± 16.14 g/m^2^. There was no significant association of LV EF, LV mass-to-EDV, ECV, or τ_*ic*_ with LGE (% by 5SD). CCC patients with ECV above the median had more severe HF symptoms (NYHA class; *P* = 0.04) and had a lower blood hematocrit (39 vs. 42%; *P* = 0.049), but did not differ otherwise in patient characteristics, as shown in Tables 1, 2. CCC patients with ECV ≥ median had higher end-diastolic and end-systolic LV volume indices and lower LV EF (Table 2). Among patients who required cardiac transplantation, available histologic analysis using Masson's Trichrome showed the evidence of extensive diffuse myocardial fibrosis, consistent with the relatively high ECV in these cases (bullseye for ECV, Figure 1).

### Association of CMR Variables With NYHA Class and Rassi Score

**Figure 2:**
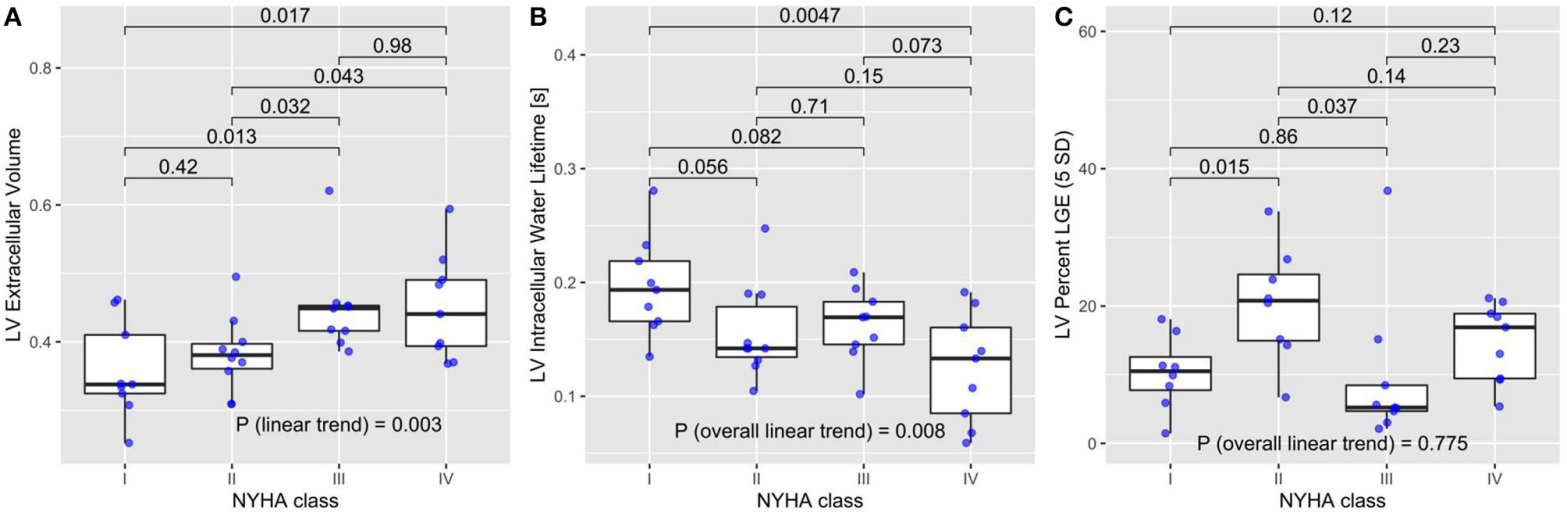
**(A)** Patients with extracellular volume (ECV) had worse heart failure symptoms assessed by the New York Heart Association (NYHA) class (*P* = 0.003 for non-parametric test for linear trend). The *P*-values at the top for NYHA classes IIIV were obtained from the pairwise comparisons adjusted by Holm's method. **(B)** The intracellular lifetime, τ_*ic*_, of water decreased with NYHA class (*P* = 0.008 for the non-parametric test for linear trend). *P*-values at the top are from were obtained from the pairwise comparisons adjusted by Holm's method. **(C)** LGE percent by 5SD did not show a significant trend with NYHA class (*P* = 0.775 for the non-parametric test for linear trend).

**Figure 3:**
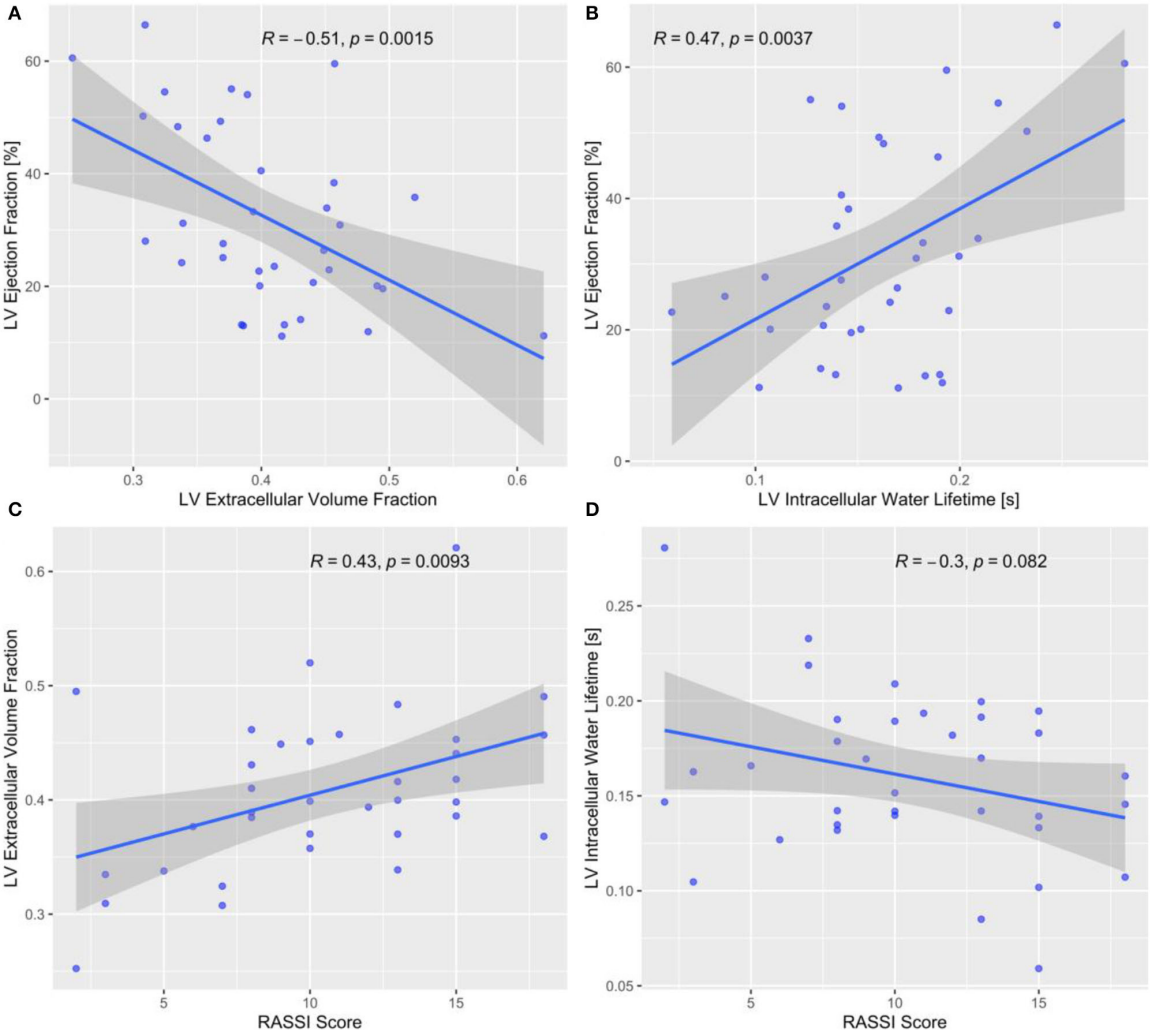
**(A)** Left ventricular (LV) ejection fraction (EF) decreased with diffuse myocardial fibrosis assessed by ECV. **(B)** LV EF decreased with intracellular water lifetime, i.e., smaller cardiomyocyte diameter. **(C)** An increasing Rassi risk score was associated with increasing LV ECV, i.e., more extensive diffuse myocardial fibrosis, and **(D)** with decreasing intracellular water lifetime.

The ECV increased overall linearly with NYHA classes (*P* = 0.007 for the non-parametric test of linear trend; Figure 2A). Pairwise comparisons of ECV between NYHA classes confirmed this increase in ECV, except for NYHA classes I vs. II and III vs. IV. The intracellular lifetime, τ_*ic*_, of water decreased across NYHA classes (*P* = 0.018 for the non-parametric J-T test of linear trend; Figure 2B). LGE estimated with the 5SD criteria was not associated with NYHA class (5SD: *P* = 0.775 for the non-parametric test for linear trend, Figure 2C). The Rassi score trended higher in the group with ECV ≥ median (12 vs. 8; *P* = 0.078 for the Wilcox test). The ECV increased significantly with the Rassi score (r = 0.43; *P* = 0.0093, Figure 3C) and τ_*ic*_ trended lower with the Rassi risk score (r = −0.3; *P* = 0.082, Figure 3D). There was no significant association between LV cardiomyocyte mass index and NYHA class (r = 0.293; *P* = 0.193) and Rassi score (r = 0.204; *P* = 0.232).

### Effects of Tissue Remodeling and LV Structure and Function

An increasing burden of LV interstitial fibrosis, assessed by ECV, was associated with a decreasing LV mass-to-EDV ratio (r = −0.52, *P* = 0.0012; Supplementary Figure 2A), while the cardiomyocyte diameter, assessed by τ_*ic*_, was correlated positively with LV mass-to-volume (r = 0.61; *P* < 0.0001; Supplementary Figure 2B). In contrast, the total LV cardiomyocyte mass was not significantly associated with LV mass-to-EDV (r = 0.23; *P* = 0.17). Myocardial ECV showed a moderate negative correlation with LV EF (r = −0.51; *P* = 0.0015; Figure 3A), and τ_*ic*_ was correlated positively with the LV EF (r = 0.47; *P* = 0.0037; Figure 3B). A higher native T1 was correlated with a lower LV EF (r = −0.43; *P* = 0.008). The difference in the correlations between LV EF and ECV, and LV EF and LGE, was significant (*P* = 0.0032 from Steiger's test for the comparison of correlations). Similarly, the difference in the correlations between LV mass-to-EDV and τ_*ic*_, and LV mass-to-EDV and LGE, was significant (*P* = 0.009 from Steiger's test for the comparison of correlations).

### Survival Analysis

**Figure 4:**
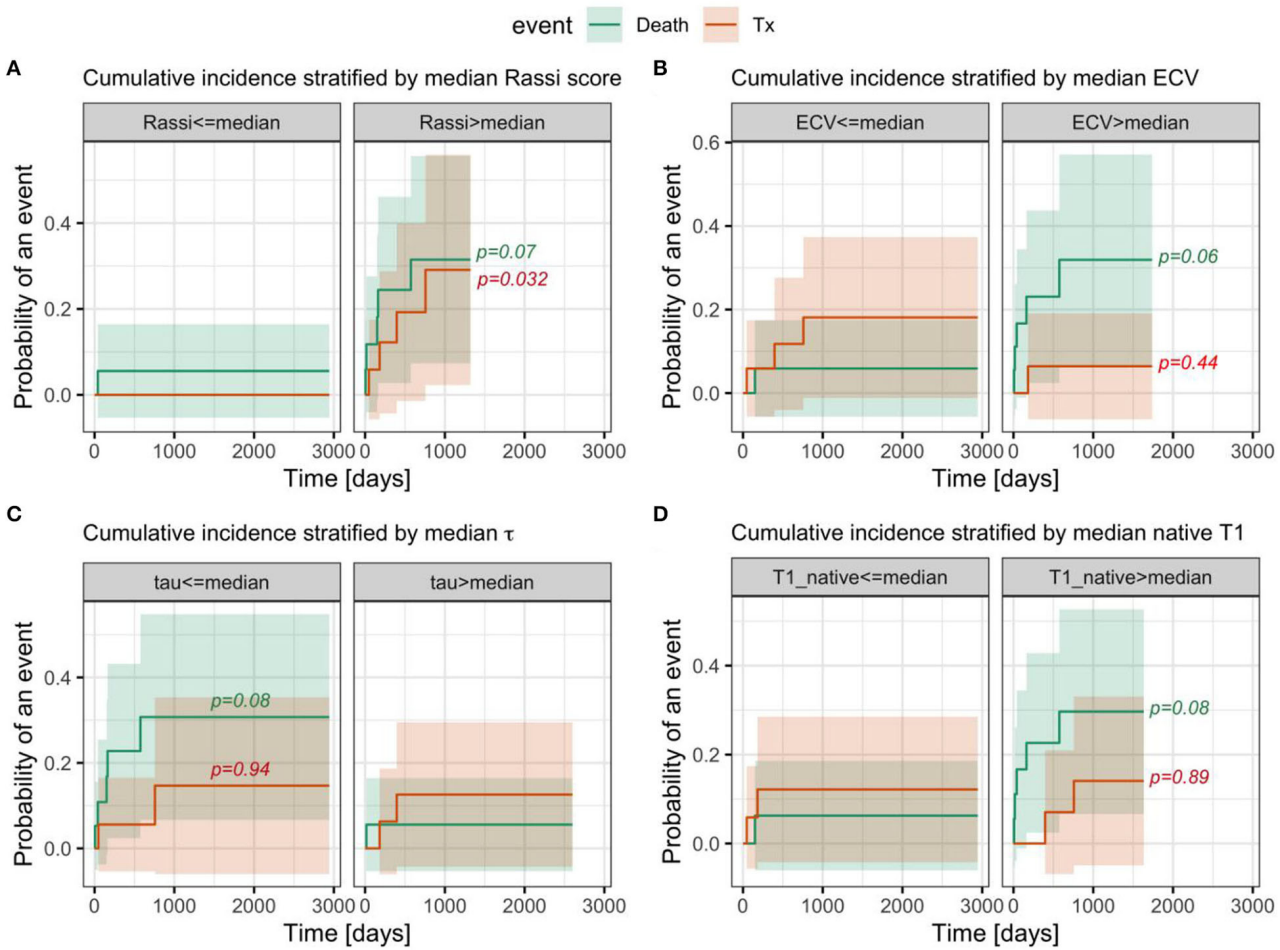
The cumulative incidence of competing risks (cardiovascular death, cardiac transplantation, and censoring) was stratified by the median **(A)** Rassi score, **(B)** ECV, **(C)** intracellular water lifetime, and **(D)** native T1, respectively. The bands around each CIF denote the 95-percentile confidence intervals. The *P*-values are from tests comparing the cumulative risk curves between strata for each event type. Of note, ECV, intracellular water lifetime, and native T1 are predominantly of prognostic value for cardiac death, rather than heart transplantation, while the RASSI score and NYHA score do not show significant differences for these competing outcomes.

Follow-up was performed between September 2012 and October 2020. During the median follow-up time of 734 days (range: 6–2,943), 10 (27%) subjects experienced HF transplantation (*n* = 4) or CV deaths (*n* = 6). The cumulative incidence of CV death trended higher with above-median ECV (ECV ≥ median of 0.40, *P* = 0.06), shorter intracellular water lifetime (τ_*ic*_ ≥ median of 0.160 s, *P* = 0.08), longer native than median T1 (native T1 ≥ median of 1,245 ms, *P* = 0.08), Rassi score ≥ median (Rassi score > 10, *P* = 0.07) (Figures 4A–D), and in patients with HF classifications NYHA III–IV compared to NYHA I–II (*P* = 0.006; Supplementary Figure 3). The cumulative incidence of cardiac transplantation was higher (*P* = 0.032) in the group with the Rassi score above its median value of 10 and in patients with NYHA III or IV (*P* = 0.031). Univariate Cox models with cardiac transplant as a competing risk identified the following significant predictors of CV death: Rassi score (heart rate [HR] = 1.37 for each 1 unit change; 95% CI = [1.06, 1.76]; *P* = 0.015), ECV (HR = 3.66 for 0.1 change, 95% CI = [1.34, 10.0], *P* = 0.012) and higher HR (HR = 1.094 for 1 bpm increase, 95% CI = [1.0283, 1.1636]; *P* = 0.0045). LV EF (HR = 0.97 for 1% decrease; 95% CI = [0.91, 1.03]; *P* = 0.28, τ_*ic*_ (HR = 0.377 for 0.1 s change, 95% CI = [0.063, 2.24], *P* = 0.283), native T1, and the extent of LGE were not associated with CV death in the respective univariate Cox proportional hazard models. To ascertain the incremental value of ECV, a multi-variable Cox model was created with the addition of ECV as a predictor to the Rassi score. An elevated ECV provided an incremental prognostic value to the Rassi score (Supplementary Figure 4).

## Discussion

The main findings of this study are that ECV and intracellular water lifetime, or cardiomyocyte diameter regression, correlate with adverse ventricular remodeling and NYHA HF classification. While cardiomyocytes undergo hypertrophy as an early response to *T. cruzi* infection, this study shows for the first time that in patients with CCC and HF symptoms, the cardiomyocyte diameter regresses. Furthermore, the expansion of extracellular space by ECV was significantly associated with the Rassi score, a validated and recognized risk score, for cardiovascular events in patients with CCC. LV remodeling contributes to the development and progression of CCC, ultimately leading to moderate to severe HF symptoms. This study identifies cellular and tissue factors associated with adverse ventricular remodeling in CCC.

Several landmark pathological studies have shown that focal fibrosis atypical for ischemia is prominent in CCC and can occur both in specific segments—often explained by the fact that they are watershed sites of the myocardium (21, 22)—and scattered diffusively (23)—possibly due to sustained *T. cruzi* infection and the associated inflammation (24, 25). This study is novel because the results indicate that *diffuse* interstitial fibrosis, as opposed to *focal* replacement fibrosis seen by LGE, relates to ventricular remodeling, HF status, and prognosis. ECV provided an incremental prognostic value to the widely accepted Chagas risk score introduced by the Rassi score (15) and could prove useful for risk stratification and treatment decisions if this finding can be validated in larger cohorts.

It has been noted from histological specimens from Chagas' patients that a dense extracellular collagen accumulation envelops each myocardial fiber and this prevents normal distension and contraction (26). The more advanced and late-stage cardiac phenotype of chronic Chagas' disease is characterized by ventricular dilation, and to a lesser degree increased cardiac mass (27), resulting in an overall decreased LV mass-to-EDV ratio. In particular, men in our CCC cohort could be classified as having eccentric LV remodeling, based on their LV mass-to-EDV ratio above the normal range. The negative correlation of LV mass-to-EDV ratio with ECV suggests that eccentric remodeling is associated in CCC with a higher burden of interstitial fibrosis.

Cardiomyocyte hypertrophy is linked to the *early* inflammatory response in Chagas' disease (28). Based on the measurements of the intracellular water lifetime, the cardiomyocyte diameters decreased with eccentric remodeling (LV mass-to-EDV ratio) and more severe HF. Cardiomyocyte hypertrophy in Chagas may, therefore, regress as these patients develop signs and symptoms of HF. In experimental models of hypertension and HF, eccentric remodeling with cardiomyocyte lengthening, and reduction of the cardiomyocyte diameter, is a slow, progressive change that begins before there are signs and symptoms of HF (29). The total LV cardiomyocyte mass index (LV mass index, multiplied by intracellular volume fraction) did not correlate with LV mass-to-EDV, suggesting that while the cardiomyocyte diameter regresses with eccentric LV remodeling, this is likely accompanied by a lengthening of the cardiomyocytes, as observed in other studies (30), resulting in a relatively stable cardiomyocyte volume or mass.

In this study, ECV was more strongly correlated with systolic dysfunction than LGE. Similarly, the marker of cardiomyocyte diameter was more strongly correlated with eccentric remodeling than LGE. Both findings suggest that ECV and intracellular water lifetime represent the characteristics of the myocardial tissue phenotype that are more closely linked to adverse ventricular remodeling and systolic dysfunction in advanced CCC than the amount of focal fibrosis measured by LGE. Ventricular dilation and a reduced cardiomyocyte diameter may be hallmarks of the late state of Chagas' disease when a systolic function is at its nadir. ECV and native T1 above its median resulted in a trend toward a significant increase in the cumulative incidence of cardiac death. These tissue biomarkers detect adverse remodeling in the myocardium-like diffuse interstitial fibrosis, measured by ECV, which has been shown to increase the risk of death in patients with HF independent of LV EF (31).

### Limitations

The current study has several limitations. First, this is a single-center observation with a small sample size. Second, the Look-Locker technique was used for T1 measurements in a single mid-LV short-axis slice rather than the modified Look-Locker imaging (MOLLI) covering multiple slices in the LV (the MOLLI software only became available after most patients had been recruited for this study). Although MOLLI and Look-Locker T1 measurements demonstrated acceptable agreement (32), direct comparison of results obtained using the Look-Locker T1-mapping method, to other more contemporary T1 mapping methods, may not be feasible. Third, NT-proBNP and high-sensitive troponin were not available. Fourth, the patients with CCC in our cohort were predominately symptomatic with >75% of cohort with NYHA class ≥ II. In addition, while the etiology of HF was attributed to Chagas' disease by clinical judgment and LGE pattern, our cohort of patients with CCC had a non-insignificant number of risk factors for CAD, and significant coronary disease was present in one patient. Indeed, our results may not be applicable to patients with CCC with different characteristics, and further studies including a more ample spectrum of patients with CCC are required. Functional assessment and classification of disease severity were based on clinical scales (e.g., NYHA) with known limitations with regard to reproducibility and their association with objective functional measures. Finally, the small sample size and the limited number of events limit the robustness of the current outcome analysis, which should be interpreted as providing exploratory hypothesis-generating evidence, suggesting that assessing the expansion of the myocardial extracellular remodeling may improve the current risk stratification of patients with CCC.

## Conclusion

The present results suggest that an increase in interstitial fibrosis measured by ECV, and reduced cardiomyocyte diameter, assessed by the intracellular water lifetime, has potential implications for the cardiomyopathy severity and prognosis of patients with CCC. This exploratory study indicates that ECV and intracellular water lifetime identify a tissue phenotype in patients with Chagas cardiomyopathy at a risk for adverse ventricular remodeling, HF, and cardiovascular events.

## Clinical Perspective

Chronic Chagas cardiomyopathy (CCC) constitutes the most life-threatening consequence of the *Trypanosoma cruzi* infection. CCC is characterized by an increase in myocardial fibrosis, and the extent of myocardial fibrosis in CCC has been associated with cardiovascular events and mortality. Cardiac magnetic resonance (CMR) imaging is a well-validated modality for myocardial tissue phenotyping with promising prognostic findings in patients with CCC. However, most studies of patients with CCC used late gadolinium enhancement (LGE) to assess focal fibrosis which underestimates diffuse fibrosis. Furthermore, other characteristics of the tissue phenotype, such as cardiomyocyte size, have not been assessed to-date. This study investigated the association between novel myocardial tissue markers with HF status and cardiovascular outcomes in CCC. A novel finding is that *diffuse* interstitial fibrosis is more strongly correlated with systolic dysfunction than LGE. Similarly, the marker of cardiomyocyte diameter is more strongly correlated with eccentric remodeling than LGE. In survival analysis, ECV was associated with CV death. These findings indicate that in patients with CCC novel, CMR-based tissue phenotype markers are related to HF status and clinical outcomes.

## Data Availability Statement

The raw data supporting the conclusions of this article will be made available by the authors, without undue reservation.

## Ethics Statement

The studies involving human participants were reviewed and approved by CAAE: 2647481920005404. The patients/participants provided their written informed consent to participate in this study.

## Author Contributions

CN, TS, LM, LS, LP, AS, WN, FF, SS, JS, EA, KM, TN, MJ-H, and ORCF made substantial contributions to conception and design, acquisition of data, or analysis and interpretation of data, drafted the article or reviewed it critically for important intellectual content, and given final approval of the version to be published. All authors contributed to the article and approved the submitted version.

## Funding

ORCF was supported by grant in research productivity (303366/2015-0) from the National Council for Scientific and Technological Development (CNPq) and by grants from the São Paulo Research Foundation (2015/15402-2, 2016/26209-1, and 2017/03708-5). TN was supported by a gift from A. Curt Greer and Pamela Kohlberg, and grants from the National Institutes of Health/National Heart, Lung, and Blood Institute under grants R01HL130539, R01HL137562, K24HL150238, and the National Institutes of Health/Harvard Center for AIDS Research under grant P30 AI060354.

## Conflict of Interest

TN has received support from Astra Zeneca, Bristol Myer Squibb, AbbVie, Amgen, and H3 Biomedicine. ORCF has received research grants and/or speaking honoraria from Amgen, Astra Zeneca, Bayer, Boehringer Ingelheim, Novartis, Takeda, and Pfizer. The remaining authors declare that the research was conducted in the absence of any commercial or financial relationships that could be construed as a potential conflict of interest.

## Publisher's Note

All claims expressed in this article are solely those of the authors and do not necessarily represent those of their affiliated organizations, or those of the publisher, the editors and the reviewers. Any product that may be evaluated in this article, or claim that may be made by its manufacturer, is not guaranteed or endorsed by the publisher.

## Abbreviations

λ_Gd_: partition coefficient for gadolinium contrast
BSA: body surface area
CAD: coronary artery disease
CCC: Chronic Chagas cardiomyopathy
CDC: The Centers for Disease Control and Prevention
CMR: cardiovascular magnetic resonance
DM: diabetes mellitus
ECV: extracellular volume
τ_*ic*_: intracellular water lifetime
EDV: end diastolic volume
EF: ejection fraction
ESV: end systolic volume
FOV: field of view
ICA: invasive coronary angiography
HCHOL: hypercholesterolemia
Hct: hematocrit
HF: heart failure
HTN: systemic hypertension
IQR: inter-quartile range
LGE: late gadolinium enhancement
LV: left ventricle
MI: myocardial infarction
MOLLI: modified Look-Locker imaging
NEX: number of excitations
NYHA class: New York Heart Association functional classification
TE: echo time
TR: repetition time.
