# Supplementary material for: Diffuse Myocardial Fibrosis and Cardiomyocyte Diameter Are Associated With Heart Failure Symptoms in Chagas Cardiomyopathy
Source: Front Cardiovasc Med. 2022 Jun 17;9:880151. doi: 10.3389/fcvm.2022.880151 (PMC9247201; doi:10.3389/fcvm.2022.880151)
Supplement: Supplementary file 1 [file Data_Sheet_1.docx]

**Supplemental Tables (online-only Data Supplement):**

**Supplemental Table 1 (online-only Data Supplement)**: Late gadolinium Enhancement pattern distribution.

| LGE Pattern | (n) | % |
| --- | --- | --- |
| Mesocardial (only) | 2 | 6 |
| Epicardical (only) | 18 | 53 |
| Transmural (only) | 0 | 0 |
| Mesocardial and Epicardical | 8 | 24 |
| Mesocardical and Transmural | 1 | 3 |
| Mesocardial and Epicardical and Transmural | 1 | 3 |
| Epicardial and Transmural | 4 | 12 |

**Supplemental Table 2 (online-only Data Supplement)**: Late gadolinium Enhancement distribution per apical, anterior, lateral, septal, and inferior segments.

| Segments distribution | (n) | % |
| --- | --- | --- |
| Apical (only) | 0 | 0 |
| Anterior (only) | 0 | 0 |
| Lateral (only) | 5 | 15 |
| Septal (only) | 0 | 0 |
| Inferior (only) | 0 | 0 |
| Apical and Anterior | 1 | 3 |
| Apical and Anterior and Lateral | 2 | 6 |
| Apical and Anterior and Lateral and Septal | 0 | 0 |
| Apical and Anterior and Lateral and Septal and Inferior | 0 | 0 |
| Anterior and Lateral | 3 | 9 |
| Anterior and Lateral and Septal | 0 | 0 |
| Anterior and Lateral and Septal and Inferior | 0 | 0 |
| Lateral and Septal | 0 | 0 |
| Lateral and Septal and Inferior | 1 | 3 |
| Septal and Inferior | 0 | 0 |
| Apical and Lateral | 14 | 41 |
| Apical and Septal | 0 | 0 |
| Apical and inferior | 0 | 0 |
| Anterior and Septal | 0 | 0 |
| Anterior and Inferior | 0 | 0 |
| Lateral and inferior | 3 | 9 |
| Lateral and septal and apical | 5 | 15 |

**Online Data Supplement**:

**Supplemental Figure 1 A-B** (online-only Data Supplement):

**Figure legend (online-only Data Supplement)**: a) R1 in measured myocardial tissue was fit was a function of R1 measured in blood using a two-site model of water exchange, with ECV, intracellular water lifetime, and native R1 outside the cardiomyocytes as variable parameters. The fit is shown as continuous red line, and prediction limits are shown by the blue shading. The intracellular R1 was assumed constant (0.9 s^-1^ for 3T). In contrast to the standard model for determining ECV, R1 in tissue shows a sub-linear increase at higher R1 values in blood when the rate of water exchange across cell-membranes becomes a bottleneck for relaxation of intracellular water. The estimates for ECV and intracellular water lifetime are shown in the upper left corner of the graph. The estimate from the fit of the extracellular native R1 is shown as dashed horizontal line. b) The equation for the myocardial R1 in the two-site water-exchange model, previously derived by Landis et al. (Landis CS, Li X, Telang FW, Molina PE, Palyka I, Vetek G, Springer CS Jr. Equilibrium transcytolemmal water-exchange kinetics in skeletal muscle in vivo. *Magn Reson Med*. 1999 Sep;42(3):467-78) c) Notation information for parameters in the two-site model of water exchange.

**Supplemental Figure 2 A-B** (online-only Data Supplement):


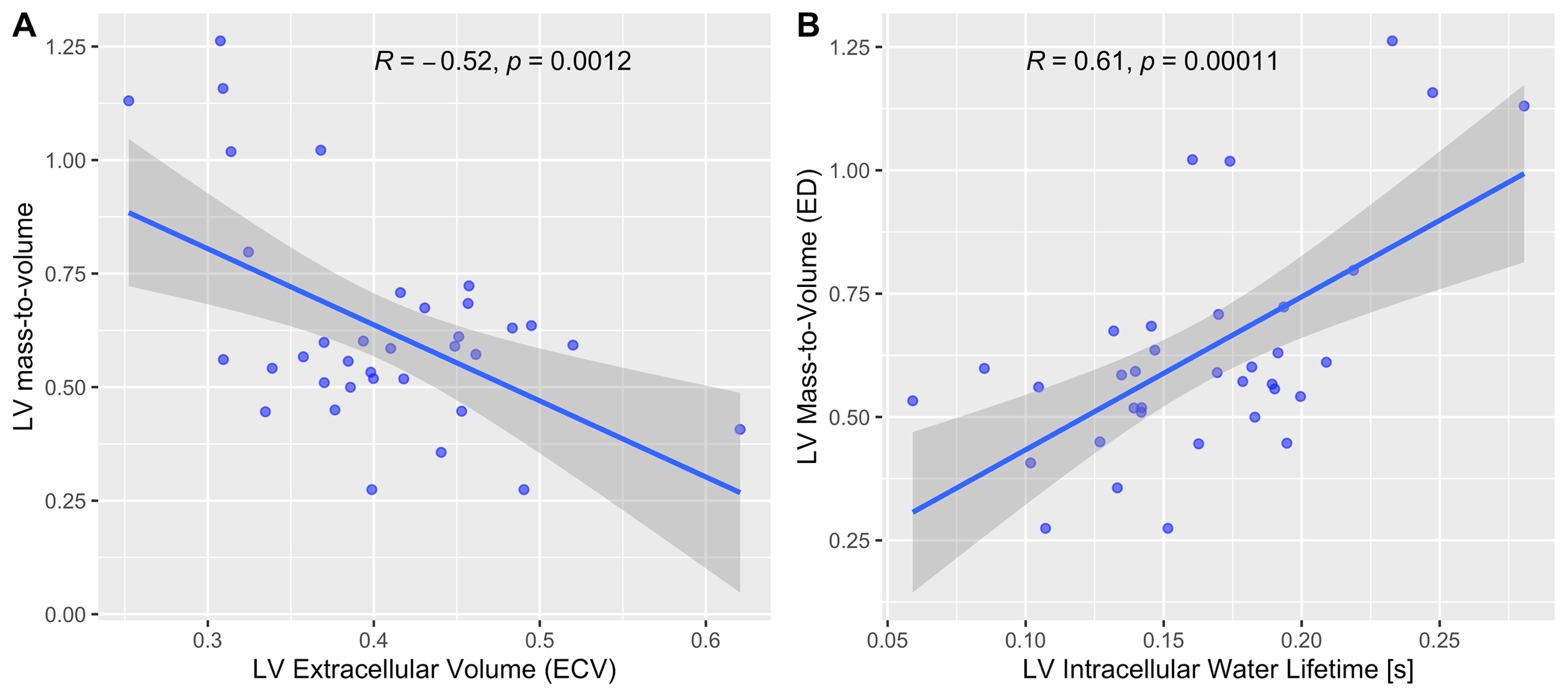


**Supplemental Figure 2 Legend (online-only Data Supplement):** Comparison of LV mass-to-volume ratio as global marker of ventricular remodeling with A: the extracellular volume fraction (ECV); B: intracellular water lifetime, a marker of cardiomyocyte diameter. Eccentric remodeling (or regression of centric remodeling) leads to a reduction of cardiomyocyte fiber diameter and lengthening of cardiomyocytes.

**Supplemental Figure 3** (online-only Data Supplement):

**
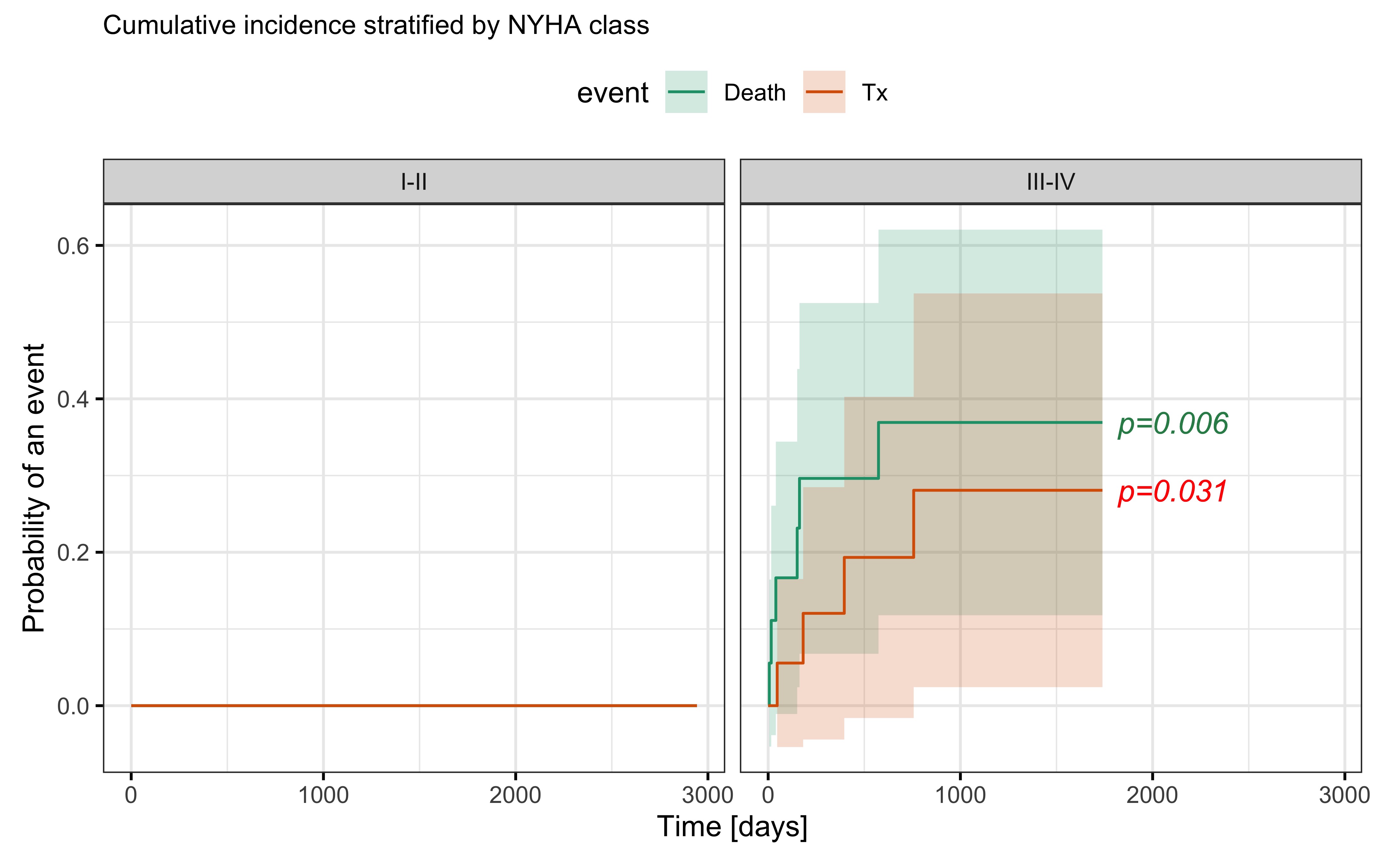
**

**Supplemental Figure 3 Legend** (online-only Data Supplement): The cumulative incidence of cardiovascular death was more likely in patients with heart failure classifications NYHA III-IV (N=18) compared to NYHA I-II (N=19, P=0.006). Similarly, the cumulative incidence of heart transplantation was higher in patients in NYHA III or IV (P=0.031). NYHA I and II were grouped together as there were no adverse events in patients with this heart failure classification. NYHA III-IV were grouped together because of the relatively low number of events.

**Supplemental Figure 4** (online-only Data Supplement):


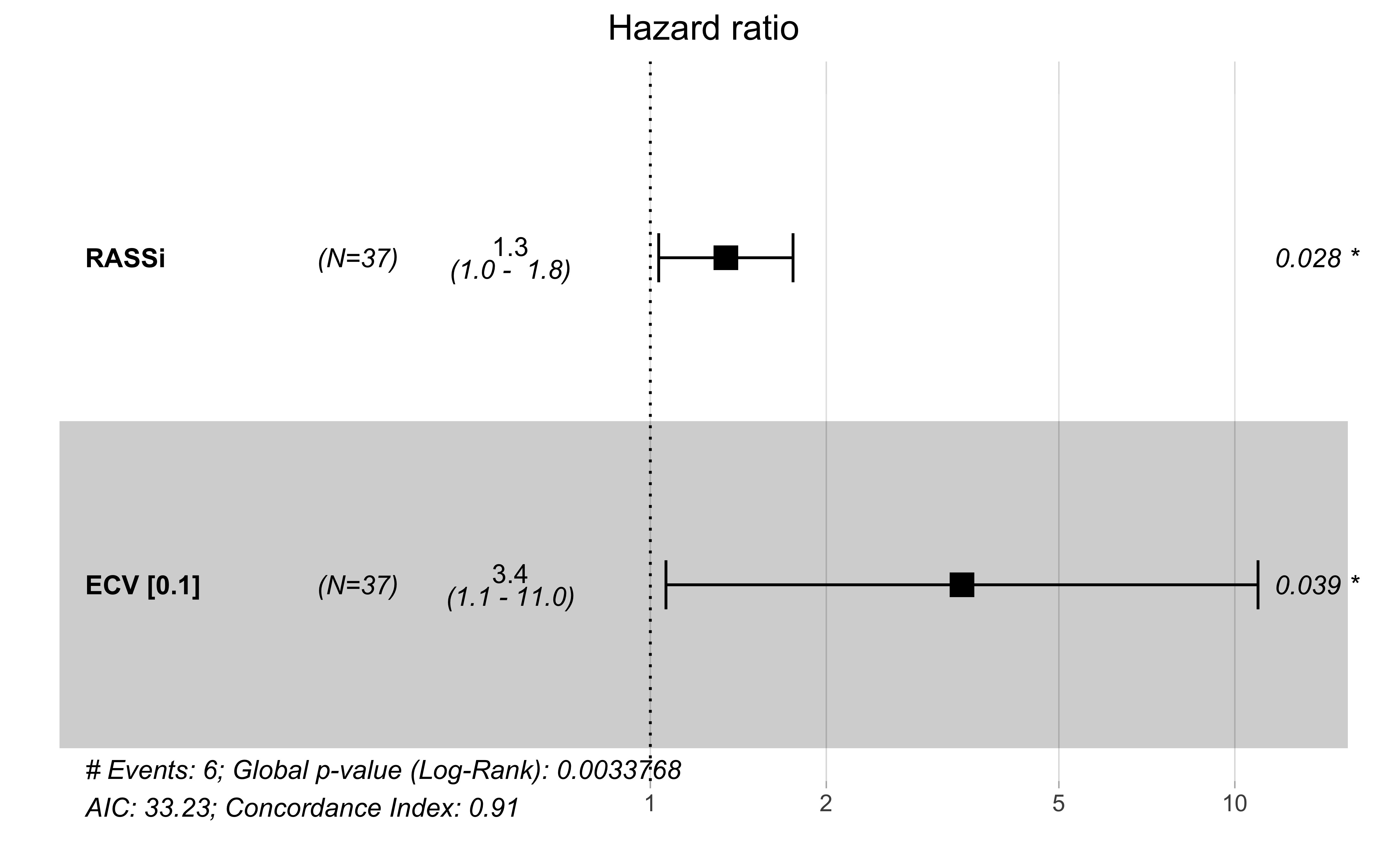


**Supplemental Figure 4 Legend** (online-only Data Supplement): The forest plots show the hazard ratios with 95% confidence intervals for a multivariate Cox proportional hazards model for prediction of cardiovascular death with adjustment for the competing risk of cardiac transplantation. The extracellular volume (ECV) remained significantly associated with CV death (P=0.021) with simultaneous adjustment by Rassi score (P=0.028).
